# Sclerosing epithelioid fibrosarcoma as a rare cause of ascites in a young man: a case report

**DOI:** 10.1186/1752-1947-2-248

**Published:** 2008-07-25

**Authors:** Philip J Smith, Beverley Almeida, Jasna Krajacevic, Barry Taylor

**Affiliations:** 1 North Cheshire NHS Trust, Warrington Hospital, Cheshire, WA5 1QG, UK

## Abstract

**Introduction:**

Sclerosing epithelioid fibrosarcoma is a rare but distinct variant of fibrosarcoma that not only presents as a deep-seated mass on the limbs and neck but can also occur adjacent to the fascia or peritoneum, as well as the trunk and spine. We report the case of an intra-abdominal sclerosing epithelioid fibrosarcoma, which to best of the authors' knowledge has not been described previously. The patient discussed here developed lung metastases but is still alive 1-year post-diagnosis.

**Case presentation:**

A 29-year-old man presented with a 2-week history of progressive abdominal distension and pain and was found to have marked ascites. A full liver screen was unremarkable with abdominal and chest computed tomography scans only confirming ascites. After a diagnostic laparotomy, biopsies were taken from the greater omentum and peritoneal nodules. Histopathology revealed a malignant tumour composed of sheets and cords of small round cells set in collagenized stroma. After further molecular investigation at the Mayo Clinic, USA, the diagnosis of a high-grade sclerosing epithelioid fibrosarcoma was confirmed.

**Conclusion:**

Sclerosing epithelioid fibrosarcoma is an extremely rare tumour, which is often difficult to diagnose and which few pathologists have encountered. This case is particularly unusual because of the intra-abdominal origin of the tumour. Owing to the rarity of sclerosing epithelioid fibrosarcoma, there is no clear evidence regarding the prognosis of such a tumour, although sclerosing epithelioid fibrosarcoma is able to metastasize many years post-presentation. It is important that physicians and pathologists are aware of this unusual tumour.

## Introduction

Sclerosing epithelioid fibrosarcoma (SEF) is a rare but distinct variant of fibrosarcoma, which mainly affects young to middle-aged adults of both sexes. Together with low-grade fibromyxoid sarcoma and hyalinizing spindle cell tumour with giant rosettes, SEF belongs to the class of fibrosing fibrosarcomas. It presents as a deep-seated mass on the limbs and neck. Approximately 50% of patients develop local recurrence and/or metastases, but systemic spread is usually delayed for 5 years or more [1]. The main histological features of this tumour comprise nests and cords of rounded cells surrounded by collagenous, hyalinized stroma. The differential diagnosis includes leiomyosarcoma, malignant peripheral nerve-sheath tumour, epithelial sarcoma, clear cell sarcoma, synovial sarcoma and epithelioid haemangioendothelioma [1,2]. Differentiation between tumours is ultimately made by immunochemical analysis. To the best of the authors' knowledge, this is the only reported case of SEF originating intra-abdominally, although other case reports have documented cases that were anatomically closely related [3,4].

## Case presentation

A 29-year-old man presented with a 2-week history of progressive abdominal distension and pain. On clinical examination, it was found that he had no signs of liver disease or lymphadenopathy, but marked, tense ascites. Although he had had a pansystolic murmur, an echocardiogram revealed only moderate tricuspid regurgitation. A full liver screen was unremarkable, which included a normal ultrasound of the abdomen, with good perihepatic blood flow. Paracentesis demonstrated an exudative nature to the ascites. Reactive mesothelial cells were noted, but not malignant cells. Abdominal and chest computed tomography scans were unremarkable, other than confirming ascites. A diagnostic laparoscopy was initially performed revealing an abnormally thickened and shortened greater omentum and mesentery. This was later converted to a laparotomy as his tumour was buried close to the mesentery of the small bowel, and it was not possible to perform a laparoscopic biopsy safely. Biopsies were taken from the greater omentum and peritoneal nodules.

**Figure 1:**
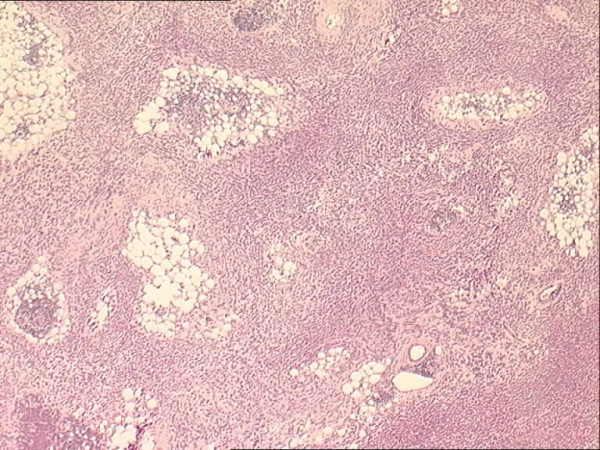
**Sclerosing epithelioid fibrosarcoma**. A tumour consisting of sheets and nests of small epithelioid cells can be seen, with minimal pleomorphism and low mitotic rate.

Histopathological analysis revealed a malignant tumour composed of sheets and cords of small, round cells set in abundant collagenous stroma (Figure 1). The cells had scanty or slightly more eosinophilic cytoplasm, and some of them showed vacuolation of cytoplasm. Nuclei were small and showed inconspicuous nucleoli and either dispersed or clear chromatin. Close to the periphery, the cells appeared more spindle-like in shape and exhibited fascicular arrangement. Mitoses were infrequent.

**Figure 2:**
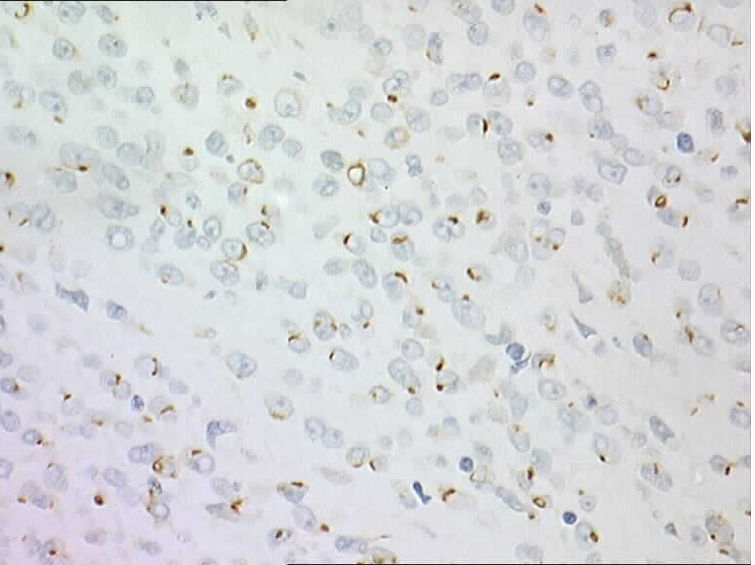
**Sclerosing epithelioid fibrosarcoma**. The tumour showed dot positivity for CKMNF 116.

The immunohistochemical analyses showed positivity for MNF116 (Figure 2) and CAM5.2 (dot-like) and equivocal positivity (occasional cells) for EMA. MiC2, B2 microglobulin and Fli-1 also showed positive reactions. Further immunohistochemical analyses showed negative reaction for LCA, CD43, S100 protein, CD34, CD31, chromogranin, synaptophysin, SMA and CD56.

Initially, the overall immunochemical profile supported the diagnosis of a primitive neuroectodermal tumour (PNET); however, the morphology was rather unusual. Differential diagnosis included SEF, intra-abdominal desmoplastic round cell tumour and epithelioid synovial sarcoma.

After further molecular investigation at the Mayo Clinic, USA, the diagnosis of a high-grade SEF was confirmed and the differential diagnoses of previously listed tumours were excluded by molecular methods: fluorescence *in situ *hybridisation for Ewing's sarcoma (EWS) locus rearrangement and reverse transcriptase, polymerase chain reaction for EWS-Fli-1 and EWS-ERG (Ewing/PNET), SYT-SSX1 and EWS-WTI (for desmoplastic small round cell tumour).

After two courses of palliative chemotherapy, treatment was discontinued at the patient's own request. Subsequently, the patient was admitted to hospital with a large pleural effusion, thought to be metastatic in nature. He remained alive 12 months post-diagnosis.

## Discussion

SEF is an uncommon tumour of deep soft tissues, usually affecting adults between 14 and 87 years of age [1]. SEF is still of clinical importance because the tumour appears benign histologically but is aggressive with full malignant potential. SEF was originally described by Meis-Kindblom et al. [2] in 1995, in their study of 25 cases, and was thought to be a low-grade fibrosarcoma capable of metastases, often many years after initial presentation.

This rare tumour usually affects the lower extremities, limb girdle, trunk and upper extremities in that order [1,2,5]. One other case report described a posterior chest wall lesion as a presentation for this tumour [5]. Previously, case reports have found that distant metastases occur to the lungs, pleura, bone, brain and lymph nodes in descending order, up to 14 years after initial diagnosis [6]. Mortality estimates have ranged from 25% in the original Meis-Kindblom et al. study [2] to 57% in other studies [1].

Owing to the tumour's rarity, the diagnosis of SEF can be difficult. The initial differential diagnosis of this tumour often includes other neoplastic lesions such as carcinoma, lymphoma and other soft-tissue sarcomas. The other sarcomas, which can assume epithelioid morphology, include leiomyosarcoma, malignant peripheral nerve-sheath tumour, epithelial sarcoma, clear cell sarcoma, synovial sarcoma and epithelioid haemangioendothelioma.

The diagnosis of SEF is ultimately established histopathologically. The tumour is characterized by an epithelioid phenotype, but in a background of dense hyalinized stroma [5]. Furthermore, diagnosis may be aided by the following criteria: small to medium cell size, clear or pale cytoplasm, cellular arrangement in cords and strands, dense collagenous stroma, rough endoplasmic reticulum and a Golgi apparatus producing collagen-secreting granules [7]. Ultrastructural evidence of fibroblastic differentiation can aid in the differential diagnosis although epithelioid appearances with marked sclerosis and infiltrating growth pattern, along with occasional immunohistochemical positivity for epithelial markers, may be highly suggestive of infiltrating carcinoma [8]. Immunochemical staining of vimentin appears to be a defining characteristic feature to aid diagnosis, although this stain was not used in this case [1,2,5-7].

## Conclusion

SEF is an extremely rare tumour, which is often difficult to diagnose and which few pathologists have encountered. This case is particularly unusual because of the intra-abdominal origin of this tumour. Owing to the rarity of SEF, there is no clear evidence regarding the prognosis for this tumour, although SEF is able to metastasize many years post-presentation. It is important that physicians and pathologists are aware of this unusual tumour.

## Abbreviations

EWS: Ewing's sarcoma; PNET: primitive neuroectodermal tumour; SEF: sclerosing epithelioid fibrosarcoma.

## Competing interests

The authors declare that they have no competing interests.

## Authors' contributions

PJS was the major contributor in writing the manuscript. BA performed the literature review associated with the case report. JK performed the histological examination of the biopsy and also gave advice on the technical aspects of the case report. BT was the overseeing consultant in charge of the case report. All authors read and approved the final manuscript.

## Consent

Written informed consent was obtained from the patient for publication of this case report and accompanying images. A copy of the written consent is available for review by the Editor-in-Chief of this journal.
